# Adipose Stem Cells and Their Interplay with Cancer Cells and Mitochondrial Reservoir: A New Promising Target

**DOI:** 10.3390/cancers16152769

**Published:** 2024-08-05

**Authors:** Ayesha Rehman, Martina Marigliano, Martina Torsiello, Marcella La Noce, Gianpaolo Papaccio, Virginia Tirino, Vitale Del Vecchio, Federica Papaccio

**Affiliations:** 1 Department of Experimental Medicine, Section of Human Histology and Embryology, University of Campania “L. Vanvitelli”, Via L. Armanni 5, 80128 Naples, NA, Italy; ayesha.rehman@unicampania.it (A.R.); martina.torsiello@unicampania.it (M.T.); marcella.lanoce@unicampania.it (M.L.N.); virginia.tirino@unicampania.it (V.T.); vitale.delvecchio@unicampania.it (V.D.V.); 2 Department of Medicine, Surgery and Dentistry “Scuola Medica Salernitana”, Via S. Allende 43, 84081 Baronissi, SA, Italy; martinamarigliano15@gmail.com

**Keywords:** ASCs, mitochondria, TME, CSCs, drug resistance, cancer therapy

## Abstract

**Simple Summary:**

Much interest has arisen around adipose-derived stem cells (ASCs) due to their multifunctional activities in the tumor microenvironment (TME). Mitochondrial dynamics and mitochondrial transfer are critical processes that promote tumor progression through fission, fusion, and transfer from stromal cells, such as ASCs. This perspective focuses on the connection between ASCs and tumor cells, leveraging the idea that inhibiting their possible pro-tumorigenic effect can interfere with these processes and limit the ability of tumor cells to survive. Unfortunately, the use of ASC/MSCs in cancer therapy has some limitations, as many variables must be considered; however, bridging the gap between preclinical studies and clinical applications could lead to new therapeutic strategies.

**Abstract:**

Adipose-derived stem cells (ASCs) significantly influence tumor progression within the tumor microenvironment (TME). This review examines the pro-tumorigenic roles of ASCs, focusing on paracrine signaling, direct cell–cell interactions, and immunomodulation. ASC-mediated mitochondrial transfer through tunneling nanotubes (TNTs) and gap junctions (GJs) plays a significant role in enhancing cancer cell survival and metabolism. Cancer cells with dysfunctional mitochondria acquire mitochondria from ASCs to meet their metabolic needs and thrive in the TME. Targeting mitochondrial transfer, modulating ASC function, and influencing metabolic pathways are potential therapeutic strategies. However, challenges like TME complexity, specificity, safety concerns, and resistance mechanisms must be addressed. Disrupting the ASC–cancer cell–mitochondria axis offers a promising approach to cancer therapy.

## 1. Introduction

**Figure 1:**
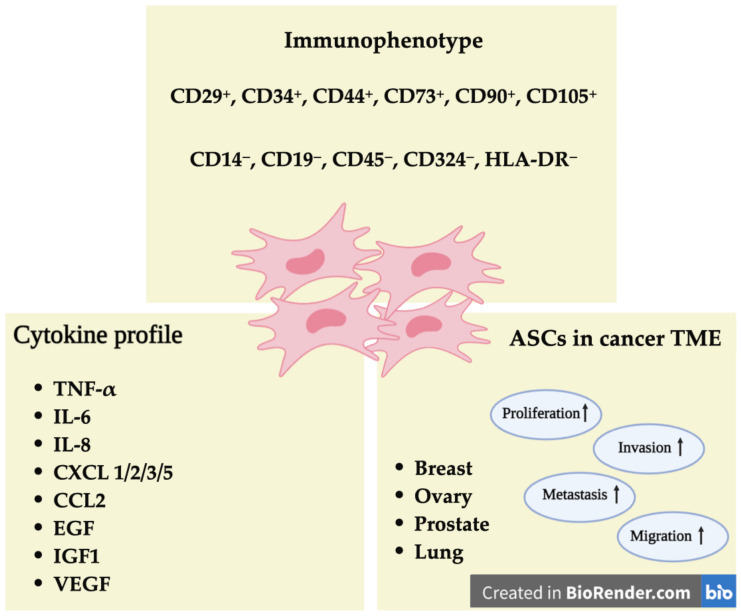
General characteristics of adipose-derived stem cells (ASCs) and their influence in the tumor microenvironment (TME) (Created by Biorender.com).

The interaction between tumors and surrounding adipose tissue has been a focus of increasing interest. Tumors, whether localized or metastatic, can be directly associated with adipose tissue. Specifically, mammary tumors engage with the adipose tissue in which the mammary gland is embedded from the onset of cancer initiation. Furthermore, several other cancers, such as those of the prostate, ovary, and lung, interact with subcutaneous or visceral adipose tissue, or with adipocytes from bone marrow in distant metastases (Figure 1) [1].

Adipose-derived stem cells (ASCs) are very important components of the adipose tissue. They have recently attracted considerable attention because they participate in the scaffolding of several tumor microenvironments (TME), playing an important role in tumor development and regulating a variety of pathways involved in paracrine signaling, direct cell–cell interactions, and immune regulation [2]. These cells show a typical immunophenotypic profile, including CD29^+^, CD34^+^, CD44^+^, CD90^+^, CD105^+^, CD19^−^, CD45^−^, CD324^−^, and HLA-DR^−^ (Figure 1) [3], with the secretion of several factors like TNF-α, IL-6, and VEGF that enhance inflammation, tumor growth, and metastasis by creating a feedback loop that promotes further cytokine release [2]. ASCs can directly interact with cancer cells through different mechanisms such as mitochondrial transfer via tunneling nanotubes and gap junctions, which can increase cancer cell survival and resistance to treatment [4]. Additionally, ASCs modulate the immune environment by inhibiting immune responses and promoting immune evasion, thereby contributing to tumor progression and metastasis (Figure 1) [5].

Interestingly, some studies have shown that ASCs are increased in obese mouse models and that their number is positively correlated with the quantity of adipose tissue. Indeed, the authors observed that obesity changes the composition, structure, and function of adipose tissue, thereby contributing to inflammation, metabolic dysfunction, and tumor aggressiveness. Consequently, the increase in obesity-associated ASCs is of crucial importance, as they participate in the creation of the TME and promote cancer progression [2,3].

Of note, cancer cells acquire mitochondria from ASCs to meet their metabolic needs [4]. Metabolic reprogramming in cancer is the process of changing metabolic pathways to meet the increasing energy and biosynthetic needs of rapidly multiplying tumor cells, allowing them to thrive, escape apoptosis, and adapt to the shifting conditions of the TME. Mitochondrial dynamics and mitochondrial transfer (MT) in cancer are critical processes that promote tumor progression, with dynamics allowing for adaptive metabolic programming via fission and fusion, and transfer from stromal cells, such as ASCs/MSCs, improving cancer cell survival and resistance to therapy by increasing their metabolic capacity [6,7,8].

Inhibition of mitochondrial dynamics and MT can interfere with these processes, limiting the metabolic plasticity of cancer cells and their ability to survive and resist therapy in the TME. Hence, this review explores how strategies such as blocking MT, modulating ASC function, and targeting metabolic pathways regulated by ASCs can significantly affect cancer treatment. By obstructing these critical interactions, researchers may be able to slow tumor development, increase the efficacy of current treatments, and improve overall patient outcomes.

This review also discusses numerous challenges that must be addressed to take full advantage of the therapeutic promise of targeting the ASC–cancer cell–mitochondria axis. These challenges involve navigating the complexities of the TME, ensuring the specificity and safety of interventions, deciphering resistance mechanisms, and overcoming translational research barriers. Addressing these issues will be important in developing medications that successfully disrupt the interactions between ASCs, cancer cells, and mitochondria, resulting in better cancer therapy results.

## 2. Main Features of ASCs: Promoting Tumor Growth

This review focuses on the pro-tumorigenic effects of adipose-derived stem cells (ASCs) within the tumor microenvironment (TME). These cells can infiltrate the TME and carry out their functions in different ways (Figure 1).

### 2.1. Paracrine Signaling

ASCs from both subcutaneous and visceral fat tissue secrete a variety of cytokines, chemokines, and growth factors. These secreted factors play roles in inflammation, angiogenesis, and the migration and proliferation of cells. Notable among these are tumor necrosis factor (TNF)-α, interleukin (IL)-6, IL-8, C-X-C motif chemokine ligand (CXCL)1/2/3/5, monocyte chemotactic and activating factor (CCL2), epidermal growth factor (EGF), insulin-like growth factor 1 (IGF1), and vascular endothelial growth factor (VEGF) (Figure 1) [2]. In fact, elevated levels of TNF-α released by ASCs establish a positive feedback loop and further stimulate ASCs to secrete multiple cytokines and chemokines that are significantly associated with enhanced metastasis and tumor growth [9]. Additionally, IL-6 released from ASCs promotes tumor progression by regulating gene expression involved in proliferation, such as a marker of proliferation Kiel 67 (MKI67) and proliferating cell nuclear antigen (PCNA) [10]. Furthermore, Kengelbach-Weigand et al. showed that ASCs secreted IL-6 and IL-8 induced tumor invasion and metastasis in breast cancer cells [11]. Moreover, Sharaf et al. observed that the ASC secretome promotes neo-angiogenesis in head and neck squamous cell carcinoma (HNSCC), an effect that they attributed to increased VEGF production [12]. Ribeiro et al. showed that adipose tissue and ASCs exposed to conditioned media from PC3 cells (prostate cancer cell line) exhibited an altered adipokine expression profile, including elevated TNF-α and IL-6 levels [13]. These factors have been linked to prostate cancer tumorigenicity and metastasis [14]. Overall, the complex interplay between ASCs and tumor cells underscores the importance of understanding how ASCs contribute to tumor progression and metastasis.

### 2.2. Direct Cell–Cell Interactions

**Figure 2:**
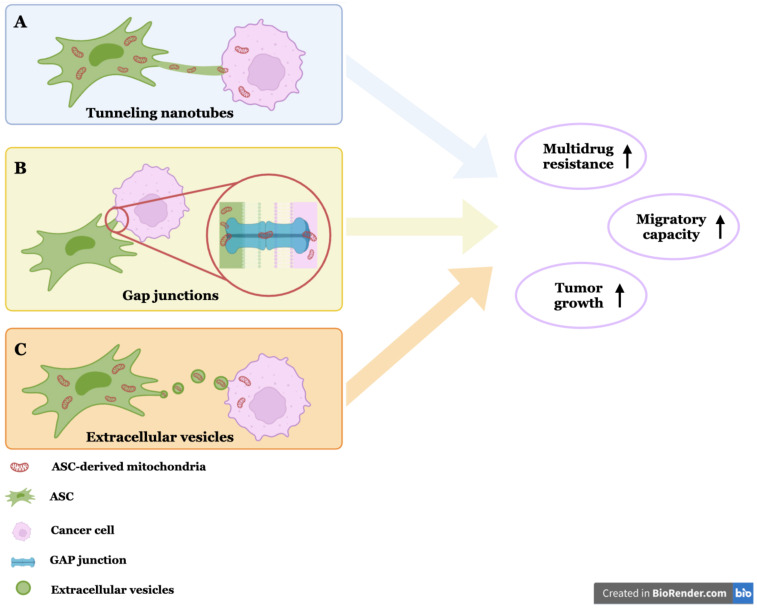
Mechanisms of mitochondrial transfer. (**A**) Tunneling nanotubes (TNTs): TNTs are nanoscale membranous channels between cells, with diameters ranging from 50 to 1500 nm, lengths of 5–200 μm, and thicknesses up to 700 nm. (**B**) Gap junctions (GJs): GJs are made from the head-to-head docking of hexameric assemblies (connexons) of tetraspan integral membrane proteins (connexins (Cx)). (**C**) Extracellular vesicles (EVs): EVs are nanosized bilayer vesicles secreted by cells and are of three types: microvesicles (MVs), exosomes, and apoptotic bodies. (Created using Biorender.com).

The interaction between ASCs and cancer cells is a pivotal factor in the progression of cancer (Figure 2). ASCs engage in physical interactions with cancer cells, facilitating the transfer of mitochondria and other cellular components via tunneling nanotubes (TNTs), gap junctions (GJs), and extracellular vesicles (EVs), thereby enhancing the metabolic activity and survival of the cancer cells. Del Vecchio et al. demonstrated that mitochondrial transfer (MT) occurs through TNTs between ASCs and various breast cancer cell lines, a process that leads to multidrug resistance due to metabolic alterations in the recipient cells [14]. Additionally, ASCs transfer their mitochondria to cancer cells through GJs, with Yang et al. identifying connexin-43 (Cx-43) as a critical component in GJ-mediated MT [15].

Burch et al. showed that ASCs can donate mitochondria via EVs to tumorigenic HEK293 cells, resulting in an increased migratory capacity of these cells [16]. Furthermore, ASCs can spontaneously fuse with breast cancer cells, resulting in a population enriched with breast cancer stem cell (CSC) markers such as CD44^+^CD24^−/low^EpCAM^+^. These findings suggest that cell fusion constitutes a direct interaction between ASCs and cancer cells [17]. Collectively, these insights into the mechanisms of ASC–cancer cell interactions underscore the significant role of ASCs in promoting tumor growth and resistance, thereby highlighting potential targets for therapeutic intervention.

### 2.3. ASC-Mediated Immunomodulation

ASCs are recognized for their remarkable ability to modulate immune responses in the TME. They exhibit low levels of major histocompatibility complex (MHC) class I and completely lack MHC class II molecules, allowing them to avoid detection by the immune system [18]. Furthermore, ASCs can influence immune responses by inhibiting lymphocyte proliferation, preventing the maturation of monocyte-derived dendritic cells (DCs), and diminishing the cytotoxic effects of natural killer (NK) cells. These effects are mediated through both direct cell–cell interactions and the secretion of various cytokines and soluble factors [19]. Notably, cancer cells have been shown to exploit the immunomodulatory properties of ASCs/MSCs to their advantage. Ramzkhah et al. observed that ASCs obtained from the breast cancer TME produce more IL-4, IL-10, and transforming growth factor beta 1 (TGF-β1), leading to higher levels of CD4^+^CD25^high^FOXP3^+^ T regulatory cells that contribute to the suppression of antitumor immunity [20]. Recently, Ramzkhak et al. demonstrated that ASCs from the breast cancer TME have also been shown to have strategic effects on peripheral blood lymphocytes (PBLs) that favor the development, growth, and metastasis of breast cancer [21]. Bharami et al. showed that ASCs have an immunosuppressive effect on NK cells by significantly reducing NK activating receptors such as NKG2D and CD69 that favored tumor immune evasion in breast cancer [22]. These studies underscore the significant role ASCs play in shaping the immune landscape of tumors and highlight their potential impact on cancer progression.

## 3. Mitochondrial Dynamics in Cancer

Mitochondrial dynamics are closely associated with tumor incidence and metastasis. Changes in the TME can also alter mitochondrial dynamics, providing a pathway for cancer adaptation.

Many cancers have dysfunctional mitochondrial dynamics, which are dependent on different ratios between mitochondrial fission-related proteins and mitochondrial fusion-related proteins [7]. The Drp1/Mfn1 expression ratio was found to be increased in hepatocellular carcinoma (HCC) tissues and was associated with a poor prognosis. For instance, Zhang et al. observed that mitochondrial fission regulator (MTFR)-2 and dynamin-1-like (DNM1L) have been associated with HCC development using GO/KEGG analysis [23]. In another study, mitofusin (MFN)-1, a mitochondrial fusion protein, was identified as a significantly downregulated candidate strongly linked with HCC metastasis. Promoting mitochondrial fusion via treatment with the glycolytic inhibitor 2-deoxy-D-glucose (2-DG) significantly suppresses the effects of MFN-1 depletion [24]. However, another study showed that blocking mitochondrial fusion via knockdown of optic atrophy-1 (*OPA-1*) and *MFN-1* attenuated oxygen consumption and cellular ATP production in tumor cells [25]. These findings highlight that mitochondrial fission and fusion are extremely intricate mechanisms that can vary significantly among different cancer types.

Apart from fission and fusion processes, dysfunctional mitophagy is also associated with tumor initiation and progression in many types of cancers. Mitophagy in response to stressors such as hypoxia and nutritional deficits aims to reduce the total number of mitochondria in the cell, thus sparing vital nutrients and limiting excessive mtROS production. Tumor cells in persistent drug tolerance (DTP) states, for example, have an OXPHOS-dependent metabolism. During the DTP state, mitophagy is activated by upregulation of PTEN-induced kinase (PINK)-1. This kinase supports DTP cells in carrying out a metabolic switch and maintaining homeostasis. Inhibition of mitophagy, either by PINK1 depletion or by the use of chloroquine, improved the initial efficacy of MAPK inhibitors, providing a new therapeutic opportunity to eradicate persister cells and prolong treatment efficacy [26].

Metabolic reprogramming is a common hallmark of cancer cells and is closely related to mitochondrial dynamics. Lu et al. found that overexpression of MTFR-2 in breast cancer cells changes glucose metabolism [27]. MTFR-2 converts oxidative phosphorylation (OXPHOS) to glycolysis in a hypoxia-inducing factor (HIF)1α- and HIF2α-dependent way. Furthermore, ROS levels are reduced during metabolic reprogramming. Anaerobic glycolysis, in fact, produces lactate, which helps reduce ROS levels, as it uses metabolic intermediates such as pyruvate. Since glycolytic enzymes are upregulated during hypoxia, inhibition of these enzymes could be a promising way to eradicate residual cells and cancer stem cells [6].

Overall, the interplay between mitochondrial fission, fusion, mitophagy, and metabolic reprogramming is crucial in cancer progression and treatment resistance. Understanding these dynamics offers potential therapeutic targets for improving cancer treatment outcomes.

## 4. Mitochondrial Transfer and Metabolic Reprogramming

The mitochondria’s dynamic nature extends beyond cellular boundaries via the mechanism of MT, which enables intercellular communication between cancer cells and their TME. Many studies have shown that cancer cells acquired mitochondria from noncancer cells to compensate for their loss of mitochondrial function [8]. The mechanisms by which cells with dysfunctional mitochondria acquire new mitochondria from other cells and the signaling pathways regulating this process are still poorly understood. Cells likely trigger this transfer in response to injury signals. TNTs are the primary cellular system for transcellular mitochondrial transfer, with other modes including EVs, GJs, cell fusion, and mitochondrial expulsion [15,28] (Figure 2). During tumorigenesis, an increase in mtDNA mutations and a marked increase in reactive oxygen species (ROS) impair OXPHOS function and cause structural and functional abnormalities in mitochondria. The transferred mitochondria can improve the bioenergetic capacity of tumor cells, promoting their survival and proliferation under stress conditions such as hypoxia or nutrient deprivation [29]. The ability of ASCs/MSCs to shelter malignant cells is clinically significant since the transfer of mitochondria or mtDNA from ASC/MSCs has been shown to restore the respiratory function of cancer cells [30,31].

### Mitochondrial Transfer Drives Tumorigenesis and Chemoresistance

The horizontal transfer of mitochondria between cancer and noncancer cells via cell–cell interactions and the production of soluble molecules and EVs are critical mechanisms that cancer cells use to evade immune surveillance and develop chemoresistance [32,33,34,35]. Pinto et al. showed that TNT-mediated MT from glioblastoma (GBM) stem cells into patient-derived tumor organoids assisted in the establishment of tumor networking with tumor microtubes, hence contributing to cancer progression and therapy resistance [36]. Some research teams have created a method called MitoCeption, which allows mitochondria from stromal cells to be transferred to tumor cells, restoring respiratory function and enhancing proliferation rates [37]. The direct transfer of mitochondria to breast cancer cells (BCCs) can boost their proliferative and invasive properties, as well as their resistance to chemotherapy treatments [4,38].

## 5. Targeting the ASC–Cancer Cell–Mitochondria Axis: Therapeutic Potential

Given the complex interactions between ASCs, cancer cells, and mitochondria, targeting this axis presents a novel and potentially effective therapeutic strategy. This therapeutic potential can be considered through several avenues.

### 5.1. Inhibition of Mitochondrial Transfer

Preventing the transfer of mitochondria from ASCs to cancer cells could reduce the metabolic adaptability and survival of cancer cells. This can be achieved by targeting mechanisms such as the formation of TNTs, GJs, and EVs. Understanding and disrupting these pathways may offer a new avenue for cancer treatment.

Since TNTs are the primary route of MT, inhibiting their formation could also be viewed as an effective therapeutic strategy. The role of taxanes and vinca alkaloids in partially obstructing MT by preventing microtubule polymerization becomes significant [5]. Additionally, inhibitors of actin polymerization, such as cytochalasin B (CytoB), cytochalasin D (CytoD), metformin, and the mTOR inhibitor everolimus, block the formation of TNTs, thereby reducing MT [39,40,41]. For instance, Del Vecchio et al. highlighted that CytoB significantly blocked MT from ASCs to BCCs in a 2D coculture model. This finding contrasts with the hybrid 2D/3D coculture, where CytoB had no significant effect, suggesting that MT is mediated by mechanisms beyond TNTs [4]. Further research is necessary to fully understand the mechanisms of TNT-mediated transfer and develop effective inhibitors.

GJ intercellular inhibitors (GJICs), such as oleamide, have shown potential in reducing metastatic foci in the liver and lungs, improving survival rates in mice injected with BCCs [42]. From this perspective, the use of medications such as mefloquine, arsenic trioxide, and carbenoxolone, which were shown to inhibit Cx43-based GJs in breast cancer bone metastasis [43], presents a promising therapeutic strategy. Notably, meclofenamate, a drug that specifically targets GJs, is currently being tested in a clinical trial in patients with recurrent or progressive brain metastasis (NCT02429570). These advancements highlight the potential of GJICs in improving outcomes for cancer patients.

Recently, a study showed that the use of exosome inhibitors like GW4869 could reduce BC chemoresistance by blocking the exosome-mediated transfer of mitochondria carrying mutant mtDNA, potentially identifying new molecular targets for more effective cancer treatment [44].

### 5.2. Modulation of ASC Function

Changing the functional state of ASCs using genetic alterations may transform their role from tumor-promoting to tumor-inhibiting. For example, increasing the release of antitumorigenic substances from ASCs may decrease tumor development. Some researchers have modified human ASCs to boost TRAIL production under TGF-β signaling via a SMAD4-controlled minimal promoter, taking advantage of the increased TGF-β expression in glioblastoma compared to normal brain tissue [45]. Lee et al. proposed a cancer treatment using osteogenic differentiated human ASC exosomes to reprogram CSCs into nontumorigenic cells, producing osteogenic-related genes and reducing drug-resistant ABC transporters and *BRCA1/2* gene expression in CSCs [46]. Similarly, Bcl-2 reduction has been achieved by packing EVs with therapeutic biomolecules such as silencing RNA (bcl-2 siRNA) and antisense oligonucleotides (ASOs), or by stripping EVs of cancer-causing circular RNA (circRNA) [47,48,49]. The use of MSC-derived EVs and nanoparticles (NPs) specifically targeting mitochondria or inducing mitochondrial damage to promote cell death and reduce metastasis has also been explored [50,51]. For example, miR-126-enriched EVs can suppress cell proliferation by regulating mitochondrial metabolism [52]. Similarly, miRNA-loaded EVs can promote cell death via the intrinsic mitochondrial pathway by suppressing antiapoptotic proteins of the Bcl-2 family [53,54].

### 5.3. Metabolic Reprogramming

Targeting the metabolic pathways in cancer cells that are influenced by MT from ASCs could reduce the survival and proliferation of cancer cells. Many studies have shown that many cancer cells can oxidize glucose via OXPHOS in their fully functioning mitochondria. Furthermore, inhibiting glycolysis does not prevent tumor formation.

Suppression of the M2 isoform of pyruvate kinase in a breast cancer model led to tumor development, as this specific isoform is responsible for the last phase of glycolysis [55]. Furthermore, blocking the conversion of lactate to pyruvate for energy production, for example, by inhibiting the enzyme lactate dehydrogenase A (LDHA), increases mitochondrial respiration in breast cancer cells, demonstrating that oxidative metabolism is still functional [56]. Tumor cells may equally depend on OXPHOS for ATP production, except for tumors with mutations in tricarboxylic acid (TCA) cycle enzyme genes. Although these enzymes are critical for mitochondrial respiration, tumors with these abnormalities continue to rely on mitochondrial activity and reprogram their metabolism to maximize the production of ROS and TCA cycle intermediates necessary for cell proliferation [57,58]. Hence, inhibiting key enzymes involved in the TCA cycle, such as isocitrate dehydrogenase, succinate dehydrogenase, and α-KG dehydrogenase, or inhibiting key players in OXPHOS, like complex I–II–III–IV, could limit the metabolic flexibility of cancer cells [59,60].

## 6. Challenges and Future Directions

Despite the promising potential of targeting the ASC–cancer cell–mitochondria axis, several challenges need to be addressed.

### 6.1. Complexity of the TME

The TME is extremely complex and dynamic, making it difficult to target specific components while preserving normal tissue function. The complexity stems from its heterogeneous composition, which includes cancer cells, immune cells, stromal cells, and signaling molecules that interact in a dynamic landscape. These interactions make it challenging to create medicines that can precisely target cancer cells while preserving the normal activities of surrounding tissues. Understanding how ASCs, cancer cells, and other stromal components interact in the TME is critical to developing effective therapies, especially because MSCs can both promote cancer cell proliferation and metastasis and inhibit tumor growth under certain conditions [61].

Therefore, a comprehensive understanding of the TME and the precise activities of ASCs is required to produce tailored medicines that can effectively combat cancer while limiting adverse effects on normal tissue function. In this context, next-generation technologies, such as single-cell sequencing of human tumors and multiomics-based technology, can play an indispensable role in providing novel insight into the ASC–cancer axis in different cancer types.

### 6.2. Specificity and Safety

A significant challenge is identifying and selectively targeting the distinct markers or pathways involved in MT without interfering with other critical cellular processes. Many MT inhibitors are not able to specifically target the mechanisms involved in MT. For instance, GJ inhibitors such as carbenoxolone are not selective for Cx43 and can inhibit sites unrelated to GJs, potentially disrupting normal cellular communication and physiological activities [62]. Moreover, the risk of long-term negative effects remains a major issue. Chronic suppression of MT may impair not just cancer cell metabolism but also normal physiological functions, resulting in unexpected toxicities. Long-term investigations and extensive clinical trials are required to determine the safety and effectiveness of the ASC–cancer axis-based therapies, but they are laborious and expensive.

Addressing these issues necessitates a multidisciplinary strategy that integrates modern molecular biology, pharmacology, and nanotechnology to develop targeted, effective, and safe medications.

### 6.3. Resistance Mechanisms

Tumor heterogeneity and the adaptive capacity of cancer cells can lead to resistance mechanisms that limit the efficacy of MT inhibitors. Tumor cells may find other mechanisms to acquire mitochondria or adjust their metabolism to live without MT, necessitating the development of combination therapies or inhibitors that target multiple pathways simultaneously. In this context, it will be crucial to identify other molecular mechanisms contributing to the elevated OXPHOS state of cancer cells and how they influence resistance to chemotherapies. Additionally, the roles of mitochondrial rebuilding, reshaping, and recycling are highly context-dependent and poorly understood [63].

Further studies focused on accurately understanding dysfunctional mitochondrial dynamics and tracking the bioenergetic and metabolic changes over time are crucial to overcoming therapy resistance in cancer treatment.

### 6.4. Translational Research

The use of ASCs/MSCs in cancer therapy is complicated, as many variables must be considered, such as the stage of the tumor and the comorbidities of the patient. Also, donor characteristics (genetics, sex, age, and health status) could play a role. Bridging the gap between preclinical studies and clinical applications remains a significant challenge, and can depend on the tumor model used, epigenetic variability, and heterogeneity of isolated ASCs/MSCs. The timing and dosage of ASC/MSC use, as well as variations in cell delivery methods and culture conditions used, influence the interaction between ASCs/MSCs and tumor cells, with implications for the therapeutic effect [64].

Consequently, for ASC-based anticancer therapy to remain useful and applicable as a mainstream cancer treatment, a more homogeneous form of ASC with specific tumor-homing ability and more effective drug delivery strategies to tumor cells is needed.

## 7. Conclusions

In conclusion, the complex connection between ASCs and tumor cells highlights an important field of research with far-reaching implications for cancer treatment. ASCs actively contribute to tumor growth via paracrine signaling, direct cell–cell contacts, and immunological regulation while also promoting mitochondrial transfer, which supports tumor cell survival and resistance to treatment. This study focuses on the prospective therapeutic potential of targeting the ASC–cancer cell–mitochondria axis, which has the potential to transform cancer treatment by interrupting these crucial relationships. However, significant problems remain, including the intricacy of the tumor microenvironment, selectivity and safety issues, resistance mechanisms, and the difficulty of converting preclinical results into clinical applications. Addressing these problems through new research and technology breakthroughs will be vital in generating successful therapies. As our understanding of these mechanisms grows, targeted techniques that disrupt the ASC–cancer cell–mitochondria axis may open new possibilities for enhancing patient outcomes and furthering cancer treatment.
